# Local Anesthesia in Dentistry

**Published:** 1914-10-15

**Authors:** 


### BIBLIOGRAPHY.

LOCAL ANESTHESIA IN DENTISTRY. This is the second re-print of Professor Guido Fischer's Third German Edition which has been edited by Dr. R. H. Riethmueller of the *Cosmos* Editorial Staff. The work is published by Lea and Febiger, of Philadelphia.

While the title and size of the book suggests a treatise on the whole subject of local anesthesia as practiced in dentistry, in reality the author practically confines his attention almost entirely to the presentation of his methods of producing local anesthesia by what he terms "Conductive" anesthesia. Briefly this implies the injection hypodermically of novocain solutions into the nerve trunks supplying sensation to the various areas of the mouth and jaws. He uses a salt solution as a solvent for the novocain, with synthetic suprarenin as a corrective. The following is the formula suggested:

| | | |
|---|---|---|
| Novocain | Grams | 1.0 to 2.0 |
| Sodium Chlorid | " | 0.5 |
| Calcium Chlorid | " | 0.04 |
| Potassium Chlorid | " | 0.02 |
| Distilled Water, Sterile | " | 100.0 |
| Synthetic Suprarenin, (1-1000) | " | 0.002 |

The formula can be had from the supply houses put up in tablet form so as to facilitate composition by the dentist.

The author takes pains to give the technic of administration with great detail. He insists that every feature of the operation shall be made under absolute surgical aseptic precautions. Much of the book is given over to detailing the character of the instrumentation including the instrumentarium. A considerable part is devoted to the anatomy of the tissues involved.

Beautiful illustrations, mostly in differential colors, are used to make clear the manner of injection to secure results without possible dangers to the tissues involved or the patient's safety. In our judgment too much reitteration of technical and clinical processes is the great fault of the book, if it has any. If a more logical and sequential scheme of treatment had been followed it would be less confusing to follow and to clearly comprehend the authors ideas. The book is remarkable for the amount of patient research that the author has put into his subject. It would be stronger, however, if he had presented his scientific material at first and by itself, and then given the technical details more concisely and logically. In other words it lacks the pedagogic method that makes a good text book for teaching purposes. For the practitioner who is interested in the subject it is an invaluable book and every dentist should have a copy, whether he follows its teaching or not, as it will help to put the whole subject of local anesthesia on a more conservative and effective basis. It will make one of our best reference books and will induce new interest and study of this valuable method of practice.

The book is finely printed and beautifully illustrated and the publishers have given us a fine specimen of professional book making.

